# Diagnostic features of a non‐pigmented eccrine poroma with a collarette: Histopathological and dermoscopic correlation

**DOI:** 10.1002/ccr3.3848

**Published:** 2021-02-09

**Authors:** Tomoaki Takada

**Affiliations:** ^1^ Sumikawa Takada Dermatology Clinic Sapporo Hokkaido Japan

**Keywords:** collarette, dermatopathology, dermoscopy, eccrine poroma, epidermal invagination

## Abstract

This is a case that emphasized the need for detailed observation of the entire lesion in dermoscopic examination. Novel dermoscopic findings within a collarette.

## Graphical abstract

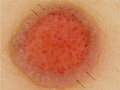

## 1 INTRODUCTION

We report a dermoscopic diagnostic clue for non‐pigmented eccrine poroma. The clue was found in a collarette, which is thought to be a nonspecific site.

Eccrine poromas (EPs) are rare benign adnexal tumors of various histological types that are dermoscopically challenging to diagnose.^1^ We present a case of non‐pigmented EP surrounded by a collarette showing a whitish‐pink ovoid nest and translucent‐brown globules with poorly visualized vessels on dermoscopy. In general, a collarette is a narrow rim of loosened keratin overhanging the periphery of a circumscribed skin lesion, which is attached to the normal surrounding skin.^2^ Because the dermoscopic examination of structures in the collarette was insufficient, a histopathological examination was performed. We identified the most common type of tumor in the dermis in continuity with the epidermis.

## 2 CASE PRESENTATION

### 2.1 Case history and examination

**FIGURE 1:**
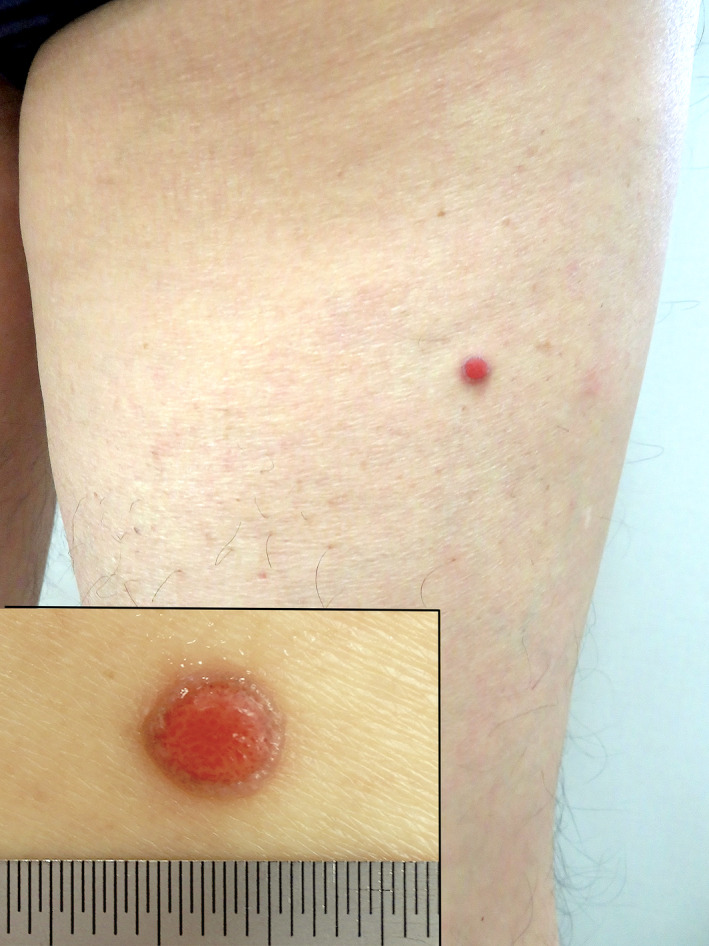
Clinical image of the eccrine poroma with collarette

A 65‐year‐old man presented with a 6.5 × 4.5‐mm, symmetrical, well‐circumscribed, non‐pigmented, deep‐red papule surrounded by an indented moat with collarette scales, on the right thigh (Figure 1). The patient had experienced trace bleeding and painful eruption 3 days prior.

**FIGURE 2:**
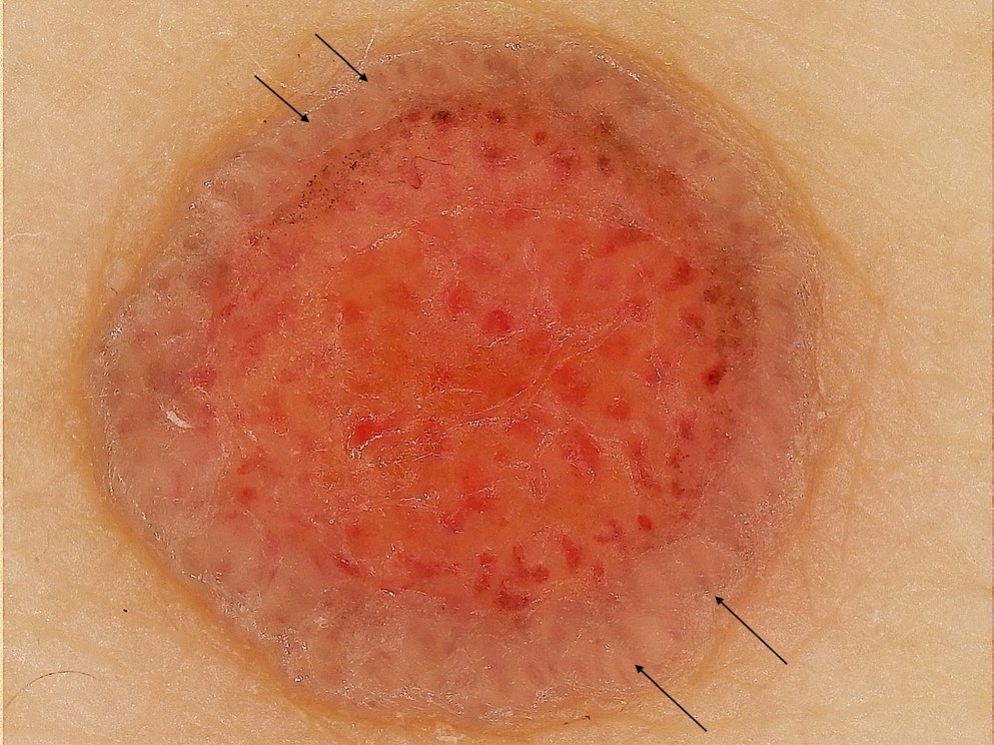
Dermoscopic image of the eccrine poroma with collarette. Milky‐red globules and chalice‐like vessels are observed. The whitish‐pink, ovoid nest and translucent‐brown globules with poorly visualized vessels in the collarette are shown (arrows)

Dermoscopic examination revealed milky‐red globules and chalice‐like vessels surrounded by a well‐circumscribed hyperkeratotic rim showing a white double–ring configuration due to thick scales. In the collarette, a whitish‐pink ovoid nest and translucent‐brown globules representing poorly visualized chalice‐like vessels were observed (Figure 2). Chalice‐like vessels were classified as branched vessels with rounded, looped, or coiled terminal endings and characteristic rounded silhouettes.

**FIGURE 3:**
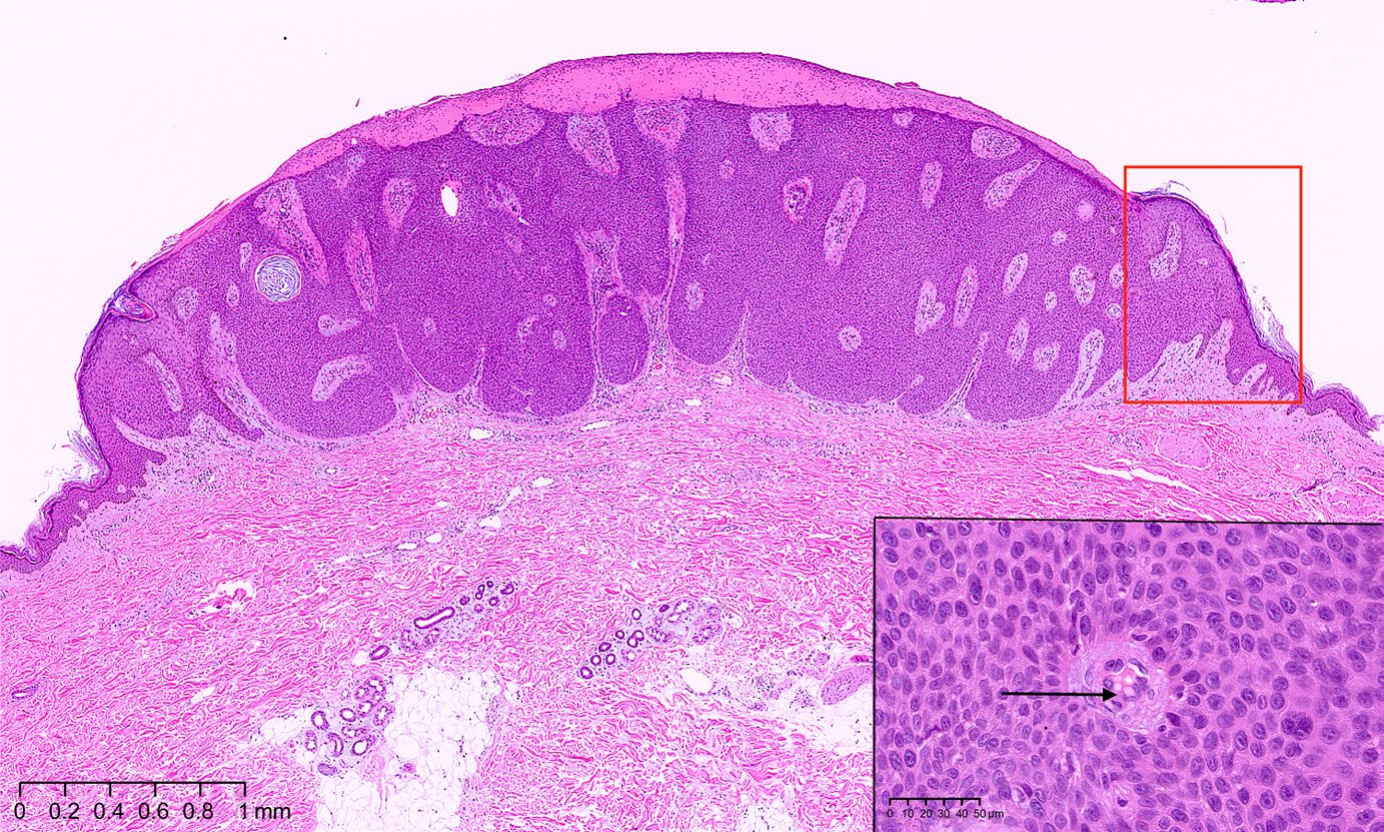
Histopathological image of the eccrine poroma with collarette, low‐ and high‐power views. The red box indicates the collarette area. The inner and outer borders of the collarette correspond to the depressed part of the epidermal invagination and the prominent part of the tumor, respectively. Small, cuboidal epithelial cells with monomorphous ovoid nuclei forming a broad, mesh‐like structure, and cytoplasmic vacuoles (arrow) are shown. Hematoxylin‐eosin stain. Original magnification, ×20; scale bar, 1 mm. Inset (black box): ×400; scale bar, 50 μm

**FIGURE 4:**
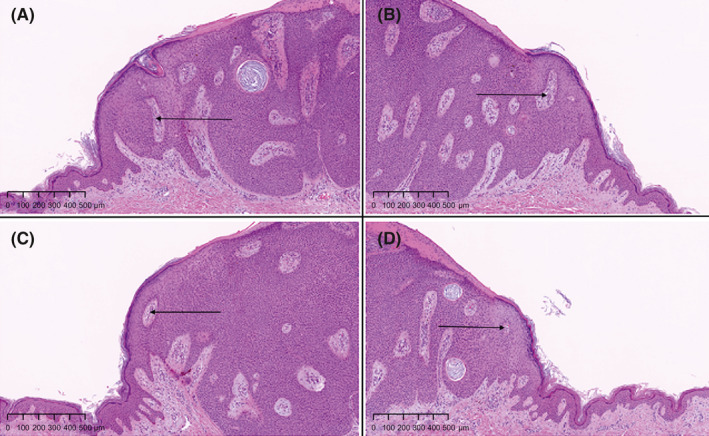
Histopathological images of four collarette parts (A–D). The invasion and anastomotic images of the tumor nest are recognized outside the epidermal invagination, and the surrounding stroma is mildly edematous with poorly visualized vessels (black arrowheads). Hematoxylin‐eosin stain. Original magnification, ×50; scale bar, 500 μm

Histological examination revealed sharply circumscribed, small cuboidal epithelial cells with monomorphous ovoid nuclei and compact eosinophilic cytoplasm. Cytoplasmic vacuoles were observed. The lesion was a poroid neoplasm mainly on the dermis, although it slightly extended to the epidermis as well. The surrounding stroma was edematous with dilated vessels of various sizes. The area of the tumor showed parakeratotic hyperkeratosis with crusting, and the granular layers had thinned or disappeared (Figure 3). At the lesion boundary, epithelial invagination was noted. Orthokeratotic hyperkeratosis, hypergranulosis, and irregular epidermal hyperplasia were observed in the skin tissues surrounding the tumor. At the boundary where epithelial invagination was noted, fissures and tearing of the thickened stratum corneum were visible. Invasion and anastomotic images of the tumor nest were identified outside the epidermal invagination, and the surrounding stroma was mildly edematous with poorly visualized vessels (Figure 4, A‐D). A tumor tissue specimen was cut vertically, and four collarette parts at both ends were examined as histopathological images.

### 2.2 Differential diagnosis, investigation, and treatment

A clinical diagnosis of EP was made, and surgical resection of the affected tissue with a 2‐mm margin of normal tissue was performed. Written informed consent was obtained from the patient for the use of the tissue samples in this study.

### 2.3 Outcome and follow‐up

The final diagnosis based on histopathological findings was EP, which was completely curatively resected. The postoperative course was encouraging, showing no evidence of a local recurrence. At present, 1 y after the operation, no findings suggesting a recurrence have been recognized and careful postoperative follow‐up is planned.

## 3 DISCUSSION

Poroma was first reported in 1956^3^ and with epithelial invagination in 1958.^4^ However, the correlations between dermoscopic findings of poroma and the pathological findings remain poorly characterized. In 2018, dermoscopic findings showed white interlacing areas around vessels, yellow structureless areas, milky‐red globules, poorly visualized vessels, and branched vessels with rounded endings. The presence of any of these five features was associated with poroma with a sensitivity and specificity of 62.8% and 82.0%, respectively.^1^ The histopathological examinations showed symmetrical, sharply circumscribed anastomosing bands of small, monomorphic cuboidal epithelial cells, with intercellular bridges in continuity with the epidermis, and no peripheral nuclear palisading. The dermal papillae appeared swollen and filled with several dilated vessels with thickened walls, surrounded by a fibrinoid edematous halo combined with peripheral lamellar fibroplasia. These findings appeared to be more pronounced on the sole than on the back. The pink‐white structureless areas and the white‐to‐pink halo were associated with specific histopathological features, comprising dermal lamellar fibroplasia and fibrinoid edema surrounding several dilated vessels, respectively.^5^ The characteristic feature was described as well‐circumscribed reddish globule/lacuna‐like structures with separation of mesh bands, which were reminiscent of frog‐egg aggregations. This characteristic feature on dermoscopy was explained by the histopathological features observed in horizontal sections: island shaped–edematous stroma containing numerous microvessels embedded in a mass of mesh‐like poroid cells.^6^ Distinct dermoscopic features appeared to be recurrent in each histopathological variant. Dermoscopy can provide important clues for the diagnosis of EP, although the final diagnosis requires histopathological examination. Because of the clinical and dermoscopic variability of EPs, surgical excision is recommended to achieve a correct diagnosis of EP.^7^

A collarette in dermatology refers to a narrow rim of loosened keratin overhanging the periphery of a circumscribed skin lesion and attached to the normal surrounding skin. The outer margin of the collarette is adherent, while the inner margin is free. In practice, however, the skin forming the collarette may be scaly or thick and ridge‐like, forming a rim around the lesion. While many disorders primarily exhibit such a morphology, it may be a secondary or a nonspecific manifestation in certain others. Dermatoses exhibiting a scaly collarette have been reported in skin tumors such as clear‐cell acanthoma, pyogenic granuloma, acral fibrokeratoma, supernumerary digit, cutaneous horn, subungual exostosis, eccrine poroma, acral fibromyxoma, stucco keratosis, and juvenile xanthogranuloma.^2^ In nonpigmented skin disorders, collarette scaling and scales are defined dermatoscopically as fragments of scales attached at the periphery and hanging like curtains, and as white homogeneous structures, respectively.^8^

The dermoscopic findings and the corresponding pathological findings in this case can be stratified according to (a) collarette parts and (b) non‐collarette parts. In collarette parts, the dermoscopic findings were: (1) whitish‐pink ovoid nest, (2) translucent‐brown dotted with poorly visualized vessels, and (3) white double‐ring translucent collarette. The pathological findings were as follows: (1) invasion and anastomotic images from the tumor nest recognized outside the epidermal invagination, (2) mild edema surrounding the stroma and absence of branched vessels with rounded endings, and (3) well‐circumscribed hyperkeratic rim showing a double‐ring configuration due to thick scales. The inner ring‐like segment was consistent with the epidermal invagination. In non‐collarette parts, the dermoscopic findings were (1) milky‐red globules, (2) chalice‐like vessels, and (3) fragments of scaly tissues. The pathological findings were as follows: (1) poroid neoplastic lesions mainly on the dermis and continuing into the epidermis; small ducts and a few cystic spaces in tumor cell clusters with edema surrounding the stroma and dilated vessels of various sizes; (2) polymorphous vascular pattern, including at least two types of vascular structures showing irregular linear vessels with semi‐elliptical endings giving the appearance of a chalice; and (3) parakeratotic hyperkeratosis with crusting at the tumor site and thinning or absence of granular layers. The characteristic dermoscopic findings^1^ allowed each of the above two sites, (a) and (b), to be diagnosed in isolation as poroma.

The dermoscopic image mainly comprised tumor with vascular structures located in the interstitial tissue, and the histopathology image further identified alterations in the spread of the tumor in the dermis, degree of inflammation, vascular hyperplasia in the interstitial tissue, and morphology of the lumens. Multiple elements were considered to combine to form a dermoscopic image. In this study, we report the diagnostic clue of dermoscopy of non‐pigmented EP in a collarette, which is thought to be a nonspecific site. Because this is a single case report, an accumulation of further studies is required. In conclusion, this was a case that recognized the need for detailed observation of the entire lesion in dermoscopic examination.

## CONFLICT OF INTEREST

None declared.

## AUTHOR CONTRIBUTIONS

TT: Collected the data and wrote the manuscript.

## ETHICAL APPROVAL

Written informed consent was obtained from the patient for publication of this case report and accompanying images.
